# *GJB2* and *GJB6* Mutations in Non-Syndromic Childhood Hearing Impairment in Ghana

**DOI:** 10.3389/fgene.2019.00841

**Published:** 2019-09-18

**Authors:** Samuel M. Adadey, Noluthando Manyisa, Khuthala Mnika, Carmen de Kock, Victoria Nembaware, Osbourne Quaye, Geoffrey K. Amedofu, Gordon A. Awandare, Ambroise Wonkam

**Affiliations:** ^1^West African Centre for Cell Biology of Infectious Pathogens (WACCBIP), University of Ghana, Accra, Ghana; ^2^Division of Human Genetics, Faculty of Health Sciences—University of Cape Town, Cape Town , South Africa; ^3^Department of Eye, Ear, Nose and Throat, School of Medical Sciences, Kwame Nkrumah University of Science and Technology, Kumasi, Ghana

**Keywords:** hearing impairment, genetics, *GJB2* and *GJB6*, Ghana, Africa

## Abstract

Our study aimed to investigate *GJB2* (connexin 26) and *GJB6* (connexin 30) mutations associated with non-syndromic childhood hearing impairment (HI) as well as the environmental causes of HI in Ghana. Medical reports of 1,104 students attending schools for the deaf were analyzed. Families segregating HI, as well as isolated cases of HI of putative genetic origin were recruited. DNA was extracted from peripheral blood followed by Sanger sequencing of the entire coding region of *GJB2*. Multiplex PCR and Sanger sequencing were used to analyze the prevalence of *GJB6*-D3S1830 deletion. Ninety-seven families segregating HI were identified, with 235 affected individuals; and a total of 166 isolated cases of putative genetic causes, were sampled from 11 schools for the deaf in Ghana. The environmental factors, particularly meningitis, remain a major cause of HI impairment in Ghana. The male/female ratio was 1.49. Only 59.6% of the patients had their first comprehensive HI test between 6 to 11 years of age. Nearly all the participants had sensorineural HI (99.5%; *n* = 639). The majority had pre-lingual HI (68.3%, *n* = 754), of which 92.8% were congenital. Pedigree analysis suggested autosomal recessive inheritance in 96.9% of the familial cases. *GJB2*-R143W mutation, previously reported as founder a mutation in Ghana accounted for 25.9% (21/81) in the homozygous state in familial cases, and in 7.9% (11/140) of non-familial non-syndromic congenital HI cases, of putative genetic origin. In a control population without HI, we found a prevalent of *GJB2*-R143W carriers of 1.4% (2/145), in the heterozygous state. No *GJB6*-D3S1830 deletion was identified in any of the HI patients. *GJB2*-R143W mutation accounted for over a quarter of familial non-syndromic HI in Ghana and should be investigated in clinical practice. The large connexin 30 gene deletion (*GJB6*-D3S1830 deletion) does not account for of congenital non-syndromic HI in Ghana. There is a need to employ next generation sequencing approaches and functional genomics studies to identify the other genes involved in most families and isolated cases of HI in Ghana.

## Introduction

Hearing impairment (HI) is a disabling congenital disease (Neumann et al., 2019), with the highest rate for age-standardized disability of life in the world (Murray et al., 2015; Vos et al., 2016). Globally, congenital HI has a prevalence of 1.3 per 1,000 population (James et al., 2018) and accounts for about 1 per 1,000 live births in developed countries, with a much higher up to 6 per 1,000 in sub-Saharan Africa (Olusanya et al., 2014). To improve the cognitive, social, speech, and language development of children living with HI, early diagnosis and intervention are recommended (Barnard et al., 2015). But in the absence of the widely used new-born screening, the age at diagnosis is usually late in Africa, e.g. 3.3 years in Cameroon (Wonkam et al., 2013). In many populations, nearly half of congenital HI cases have a genetic etiology, of which 70% are non-syndromic (Bademci et al., 2016; Sheffield and Smith, 2018). Among non-syndromic (NS) HI, nearly 80% of the cases are inherited in autosomal recessive (AR) mode (Wu et al., 2018; Zhou et al., 2019). To date, more than 98 genes have been identified, in ∼170 NSHI loci mapped (Hereditary Hearing Loss Homepage; http://hereditaryhearingloss.org/). Nevertheless, in many populations of European and Asian descent, pathogenic variants in *GJB2* (connexin 26 gene) and *GJB6* are major contributors to autosomal recessive NSHI (ARNSHI) (Chan and Chang, 2014), with the *GJB6*-D13S1830 deletion identified in up to 9.7%, as the second biggest genetic etiology of NS deafness in the European populations (del Castillo et al., 2002; del Castillo et al., 2003).

The prevalence of *GJB2*- or *GJB6*-related NSHI is very low in most sub-Saharan African populations (Gasmelseed et al., 2004; Kabahuma et al., 2011; Bosch et al., 2014; Javidnia et al., 2014; Lasisi et al., 2014). Of interest, previous studies have shown that a common founder mutation accounted for about 16.2% of congenital HI was p.R143W in a random sample of Ghanaians affected by hearing loss (Hamelmann et al., 2001). To our knowledge, the contribution of connexin 30 to HI, and the carrier frequency of the *GJB2* mutation in non-affected individuals has not been studied in Ghana (Adadey et al., 2017). In the present research, we aimed to investigate the putative environmental causes of childhood HI, and revisit the contribution of *GJB2*, and to investigate *GJB6* mutations in carefully selected samples of families segregating HI, and in isolated cases of putative genetic origin, as well control populations non-affected by HI, in Ghana.

## Methods

### Patient Participants

Hearing impaired patients were recruited from 11 schools for the deaf following procedures reported previously in Cameroon (Wonkam et al., 2013). Briefly, individuals with severe HI diagnosed before 15 years of age were enrolled in this study. For all participants, detailed personal and family history were obtained, and the medical records reviewed by a medical geneticist and an ENT specialist, and relevant data extracted, including three-generation pedigree and perinatal history. If required, a general systemic and otological examination and audiological evaluation were performed, including pure tone audiometry or auditory brain stem response test. We followed the recommendation number 02/1 of the *Bureau International d’Audiophonologie (BIAP*), Belgium, to classify the hearing levels (Bureau_International_d’Audiophonologie, 1997; Wonkam et al., 2013). After consultation with the medical geneticist, individuals with syndromic deafness underwent additional assessment, when possible. As previously reported (Wonkam et al., 2013), HI was defined as: 1) acquired when associated with a putative environmental factor such a clinical evidence of meningitis; 2) genetic when at least two cases were reported in the same family without obvious environmental cause, in case of consanguinity, in case of presence of dysmorphism or developmental problems in addition to HI, or in case of a well-defined syndrome in clinically suspected; 3) of unknown etiology if either an environmental or a genetic origin were not clearly established.

### Control Participants

A total of 145 control participants without any personal or familial history of HI was randomly recruited in Ghana, from an apparently healthy individual, during a tuberculosis screening study.

### Molecular Methods

Peripheral blood was used for genomic DNA extraction, following the instructions on the manufacturer [QIAamp DNA Blood Maxi Kit. ^®^ (Qiagen, USA)], in the Laboratory of the Department of Biochemistry, University of Ghana, Accra, Ghana.

Previously reported, primers for the *GJB2* genes were evaluated using BLAST^®^ and and other Softwares as recommended (Bosch et al., 2014). The entire coding region of *GJB2* genes (exon2) was amplified, followed by sequencing using an ABI 3130XL Genetic Analyzer^®^ (Applied Biosystems, Foster City, CA), in the Division of Human Genetics, University of Cape Town, South Africa.

Detection of del (*GJB6*-D13S1830) was performed using the method and primers described by del (del Castillo et al., 2002; del Castillo et al., 2003). The entire coding region of *GJB6* was amplified using the method described by (Chen et al., 2012). The PCR results were validated by Sanger sequencing of 10% of the sample.

### Data Analysis

Descriptive statistic and non-parametric test were used for comparisons. The level of significance was set at 5%.

## Results

### Sex, Age of Onset of Hearing Impairment

**Figure 1:**
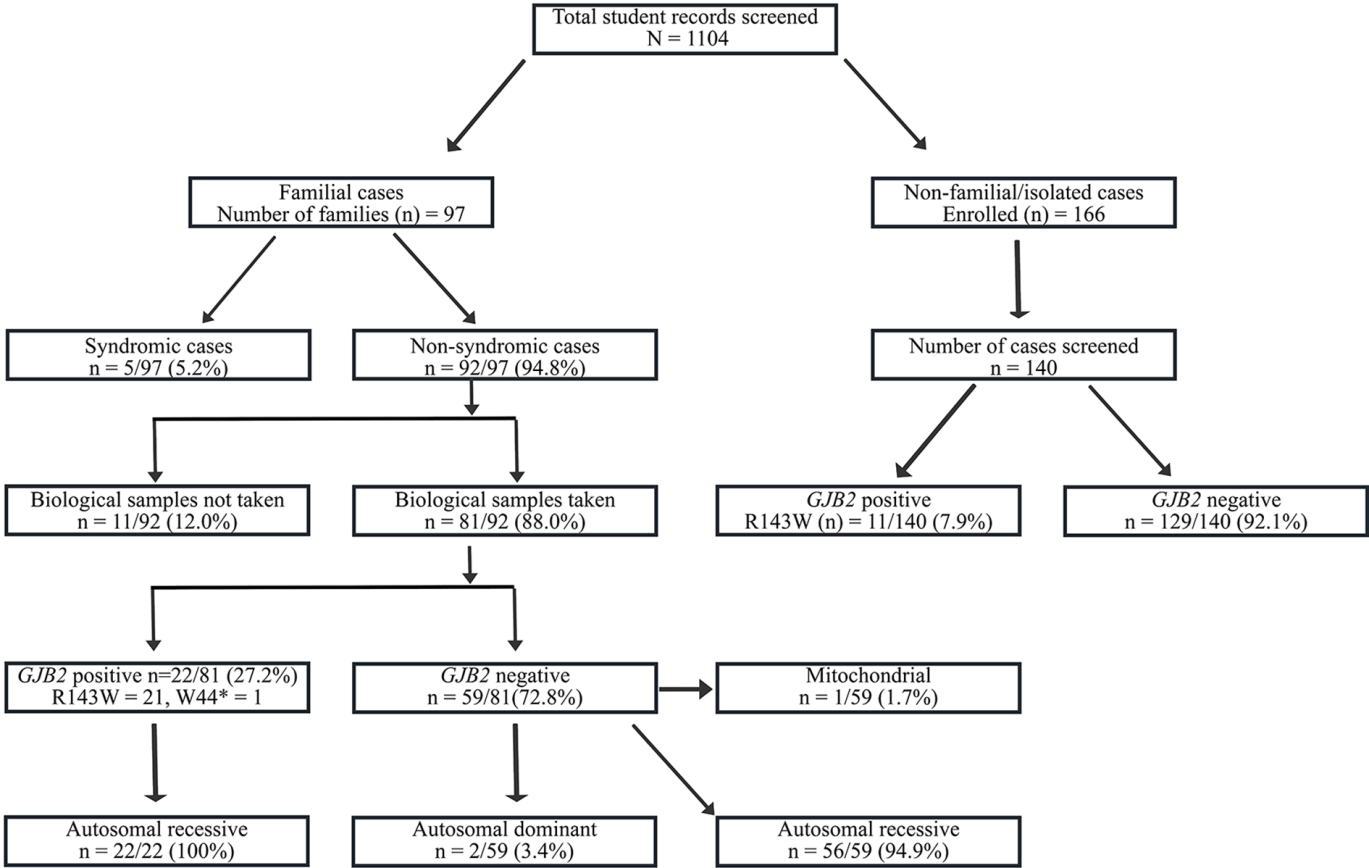
Flowchart of the recruitment and Molecular analysis of Hearing Impairment cases in Ghana. *GJB2*-R143W mutation, previously reported as founder a mutation in Ghana accounted for 27.2% (22/81) of familial, and in 7.9% (11/140) of non-familial non-syndromic congenital HI cases.

A total of 1,104 participants was evaluated (**Figure 1**). The male/female ratio was 1.49 (660/444). Most deaf participants (59.6%) had their first comprehensive HI medical test between the ages of 6 to 11 years (**Table 1** and **Figure S1A**). The median age of the students at the first medical diagnosis was 9.0 years, within a range of 2 to 22 years. The majority had pre-lingual HI (68.3%, *n* = 754; **Figure S1B**), of which 92.8% were congenital.

**Table 1:** Age at diagnosis and onset of HI.

Age of onset	Number of cases, n (%)
Prelingual (before 2 years old)	754 (68.3%)
Perilingual (between 2 and 4 years)	69 (6.3%)
Post-lingual (after 4 years)	281 (25.5%)
Total	1,104
Age at first diagnosis	Number of cases, n (%)
0–5	157 (14.2%)
6–11	658 (59.6%)
12–17	258 (23.4%)
18–23	31 (2.8)
Total	1,104

### Audiometric Characterization of HI

Analysis of the students’ medical data indicated that 642 out of the 1,104 students had a comprehensive HI test (otoscopic ear examination, pure tone audiometry, and/or tympanometry), which characteristics are described in **Table S1**. Nearly all the participants had sensorineural HI (99.5%; *n* = 639). Only 1 and 2 students had conductive and mixed HI, respectively.

### Major Etiologies of Childhood HI in the Study Population

The flowchart of the cohort is described in **Figure 1**, and the major cause of HI are displayed in **Table 2**. A lower frequency of infectious causes of HI was observed in our present study compared with other studies from sub-Saharan Africa (**Table 2**). Convulsion (with undetermined medical cause) was the most common cause of post-lingual HI followed by cerebrospinal meningitis (C.S.M.). Other diseases such as cerebral/complicated malaria, otitis media, and mumps, were also reported as causes of post-lingual HI (**Figure S2**). Over 60% of the students had congenital HI of unknown origin (**Figure S2**).

**Table 2:** Comparison of our results to other studies in developing African countries.

Country	The Gambia	Nigeria	Sierra Leone	Ghana	Cameroon	Present study
Year of publication	1985	1982	1998	1988	2013	2018
Reference	(McPherson and Holborow, 1985)	(Ijaduola, 1982)	(Wright, 1991)	(Brobby, 1988)	(Wonkam et al., 2013)	
Number of patients	259	298	354	105	582	1104
Hereditary	8.1%	13.1%	–	–	14.8%	21.3%
Meningitis	30%	11%	23.9%	8.5%	34.4%	3.9%
Measles	1.9%	13%	4.1%	30%	4.3%	0.9%
Rubella	1.5%	2%	–	3.8%	0.5%	0.2%
Mumps	–	3%	16.7%	3.5%	2.1%	0.5%
Ototoxicity	–	9%	20.8%	–	6%	–
Prematurity	–	–	–	–	0.9%	0.5%
Neonatal jaundice	–	5.7%	–	1.9%	1.4%	0.3%
Head trauma	–	–	–	–	0.3%	1.5%
Other illnesses	–	–	–	–	–	10.8%
Unspecified illness	–	–	–	–	–	6.3%
Unknown	54.4%	41.2%	34.8%	40%	32.6%	53.8%

### Familial HI With Possible Patterns of HI Inheritance

We identified 97 families segregating hearing Impairment, in 21.4% of the students. In these families, 50.9% (235/461) of children were living with HI, with an average family size of 6.9. Most of these familial cases were non-syndromic (92/97). The pedigree analysis of the non-syndromic familial cases suggested autosomal recessive inheritance in 96.7% (89/92), with only 2 families exhibiting a pattern compatible with a non-syndromic autosomal dominant inheritance. One family exhibited a mitochondrial pattern of inheritance.

**Figure 2:**
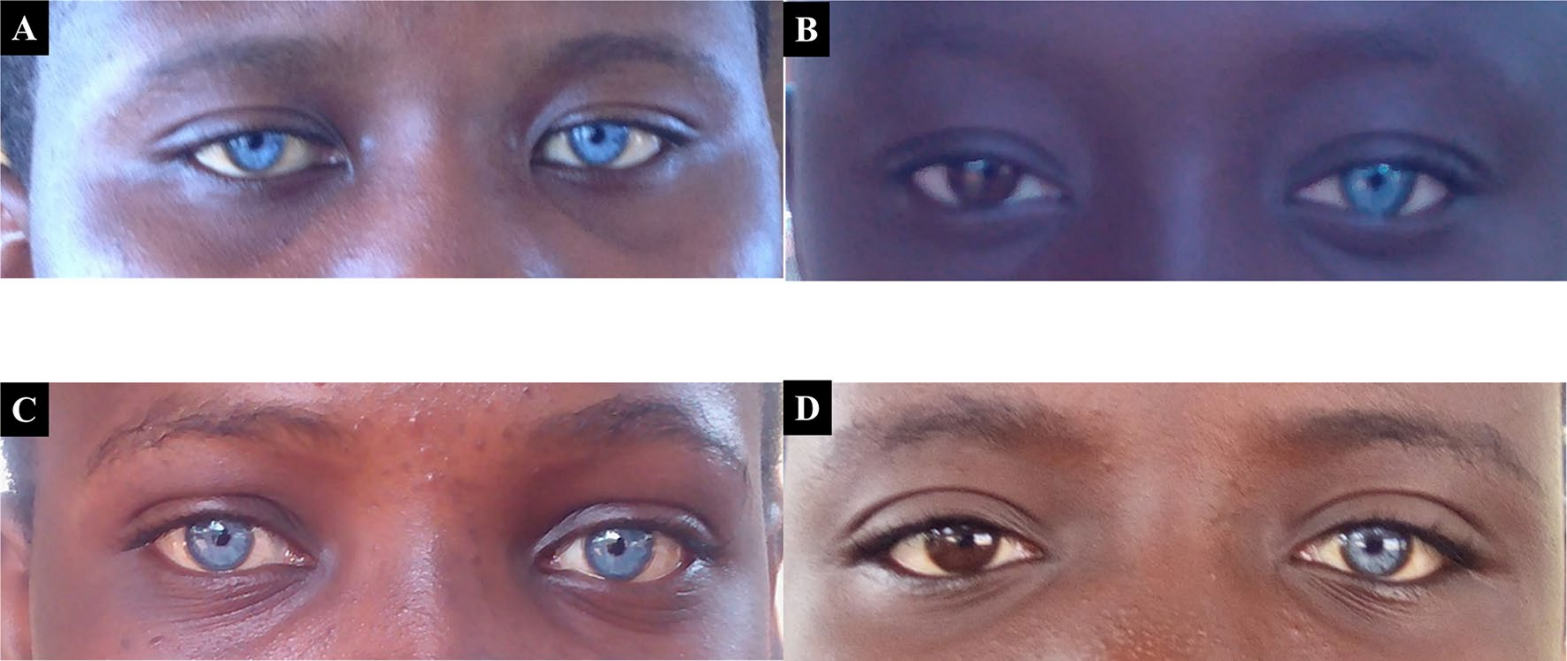
Probands with both Waardenburg syndrome, that associate variable degree of hearing impairment, and eyes/skin decoloration. Panels **(A)** and **(C)** represent patients expressing the typical bilateral striking blue eyes phenotype of Waardenburg syndrome, while **(B)** and **(D)** represent asymmetrical heterochromia, with patients expressing the phenotype in only one eye.

Waardenburg syndrome, an autosomal dominant condition, was the obvious syndromic and familial condition identified in 5.1% (5/97) of familial cases, with variable expression of heterochromia in affected members (**Figure 2**).

### Molecular Analysis Result of *GJB2* and *GJB6*

A total of 81 families segregating non-syndromic hearing loss were molecularly investigated. Although samples were not collected from Adamarobe, the ‘Deaf village,’ 27 out of the 81 HI families screened for *GJB2* and *GJB6* were from the Eastern Region of Ghana (**Table S2**) where the ‘Deaf Village’ is located (Kusters, 2012).One individual from each family was sequenced for *GJB2* mutation and we found a pathogenic mutation in 27.2% (22/81) with *GJB2*- R143W in the majority (21/22) in the homozygous state (**Table 3**); *GJB2* p.W44* mutation in one case, in the homozygous state.

**Table 3:** *GJB2* mutations among 365 previously studied and 97 Ghanaians families with profound sensorineural hearing impairment.

Nucleotide	Amino acid	Number of affected individuals			
		Previously reported (Hamelmann et al., 2001)	Our current report		
			Familial cases	Isolated/Non-familial cases	Controls
35 insG	Frameshift	1(0.3%)	–	–	–
236T→C	L79P	1(0.3%)	–	–	–
427C→T	R143W	59 (16.2%)	21 (25.9%)	11 (7.9%)	2 (1.4%)
533T→C	V178A	2 (0.6%)	–	–	–
551G→A	R184Q	1(0.3%)	–	–	–
589G→T	A197S	1(0.3%)	–	–	–
608TC→AA	I203K	1(0.3%)	–	–	–
641T→C	L214P	1(0.3%)	–	–	–
131G > A	W44*	–	1 (1.2%)	–	–

In non-familial non-syndromic cases, *GJB2*-R143W mutation was found in 7.9% (11/140) patients (**Figure 1**). The control population had 2 out of the 145 individuals with mutation *GJB2*-R143W in the heterozygous state.

No *GJB6*-D3S1830 deletion was identified in the samples screened.

## Discussion

The present report in the most compressive study of the cause of childhood HI in Ghana. Moreover, we investigated for the first time, the prevalence of *GJB2* mutations in a non-affected group of individuals from Ghana.

In this study, we observed HI in more boys than girls, although gender has not been reported as an associated factor that predisposes children to the development of HI (Foerst et al., 2006; Le Roux et al., 2015). This may be due to the fact that more boys enroll in the schools for the deaf compared to girls, especially in resource-limited regions. In often cases, boys with disability have more priority to formal education compared to girls (Groce, 1997; Nagata, 2003; Rousso, 2015). Although “female protective model” is not common to HI studies, it has been proposed by some researchers to explain the higher prevalence of genetic disorders in males compared to females (Jacquemont et al., 2014; Werling and Geschwind, 2015). According to this model, females have a higher rate of possible gene disruption but are mostly not associated with genetic disorders compared to males (Jacquemont et al., 2014).

Hearing impairment screening aims at detecting permanent HI at early developmental ages for the appropriate intervention (Sarant et al., 2008; Ching et al., 2017; Ma et al., 2018). There is no universal newborn HI screening program in Ghana explaining the late diagnosis, as most of the study participants had their first comprehensive hearing test at the school age, thus 6–9 years of age. However, parents/guardians of these children gave the information on the onset of the condition. The late diagnostic of HI in Ghanaian children is partly tied to the limited number of hearing assessment facilities (Waller et al., 2017). In addition, the majority of the HI students were living in remote rural settlements often with unmemorable roads and hence the difficulty of having access to quality health care.

Post-lingual HI in Africa is often caused by environmental factors (Wonkam et al., 2013). Similar to other reports, complicated malaria, cerebrospinal meningitis, and convulsion (with undetermined cause) were identified from our study as major environmental factors that contribute to post-lingual HI in Ghana (**Table 2**). There was a high number of congenital cases reported in our study which may account for the reduced frequency of infectious causes of HI in our study compared to other studies from Africa. Nonetheless, the identified environmental factors can be prevented by good health care systems as well as preventive health care practices. It is therefore important that governmental policies should be implemented to minimize childhood morbidities which will eventually reduce the prevalence of post-lingual HI.

Pre-lingual hearing impairment was common in our study population which agrees with other findings (Chibisova et al., 2018). Majority of pre-lingual HI are congenital and are usually caused by genetic factors (Wonkam et al., 2013; Behlouli et al., 2016). Waardenburg syndrome was the most common syndromic HI identified among the congenital cases in line with other African data (Noubiap et al., 2014).

Mutations in *GJB2* were investigated in Ghana 18 years ago and identified a common founder mutation p.R143W (Hamelmann et al., 2001). The present study revisited the contribution of *GJB2* mutations and confirm the particularly high proposition of the founder mutation in more than ¼ of families segregating HI. This is much higher than what was previously reported (18%) due to the stringent selection of familial cases in the present study. Majority of the families with HI and families positive for the founder mutation were from the Eastern Region of Ghana. It is from this Region that a high prevalence of congenital HI was reported and hence the name “Deaf Village” (David et al., 1971; Kusters, 2012). There was a relatively high proportion of *GJB2* mutations among the isolated case of putative genetics origin. This is an indication of the urgent need to implement this *GJB2*-p.R143W testing in patients with HI clinical practice in Ghana. The p.R143W mutation has also been reported in patients with HI in Japan (Zheng et al., 2015; Kasakura-Kimura et al., 2017), South Korea (Kim et al., 2016), and China (Luo et al., 2017). In addition, we report a variant previously described as Mayan: founder *GJB2* nonsense mutation (p.W44*) in a Ghanaian family. *GJB2* p.W44* mutation is the most common *GJB2* pathogenic variant in Guatemala deaf populations and was also reported in Mexico (Martínez-Saucedo et al., 2015). Ghana is an African exception, as most studies in Africa have not identify *GJB2* as a major cause of HI in sub-Saharan African populations (Lebeko et al., 2015; Wonkam, 2015).

This is the first study to investigate *GJB6*-D13S1830 mutation or coding region variations in Ghana, and we found no mutation, which is in line with previous African data (Bosch et al., 2014; Wonkam et al., 2015). Equally, *GJB6*-D13S1830 deletion was not found in populations from China (Jiang et al., 2014), India (Padma et al., 2009), Turkey (Tekin et al., 2003), and among African American and Caribbean Hispanics (Samanich et al., 2007). Therefore, the present data further support the hypothesis that the *GJB6*-D13S1830 deletion is a founder mutation (del Castillo et al., 2003).

The study also indicates more than 2/3 of families with HI are eligible for next-generation sequencing, due to the highly heterogeneous genetic nature of NSHI and the low proportion of families solved with single gene approach applied in this study. Nethetheless, the study did not exclude intronic variants in *GJB2*, that is a possible limitation. Future research should either use high-throughput sequencing platforms to investigate known genes (Shearer et al., 2010; Lebeko et al., 2016), or whole exome sequencing that will allow identification of novel genes (Diaz-Horta et al., 2012). Indeed, based on the identification of specific inner ear transcripts, it is estimated that more than 1,000 NSHI genes are still to be identified (Hertzano and Elkon, 2012).

To contribute towards the reduction of HI incidence in Ghana, policy-makers must consider integrating newborn screening for HI into the health care system such that every child is screened for both genetic and acquired HI at birth. Early detection of the condition may lead to early intervention (Copley and Friderichs, 2010) which will eventually reduce the public health impact of this condition.

## Conclusion

The study showed that environmental factors remain a major cause of Hearing impairment in Ghana. The study confirms that Connexin 26 (*GJB2*) mutations are the most common cause of familial non-syndromic HI in Ghana, an exception in sub-Saharan Africa where mutations in *GJB2* in HI patients is generally close to zero. *GJB2* p.R143W founder mutation accounted more > 25% of familial cases and close to 8% of isolated cases of putative genetic origin and should be considered in for implementation in clinical practice, particularly after newborns screening for HI. The frequency of *GJB2* p.R143W founder mutation in the general population without personal and familial was relatively high: 1.4%. The study did not find any *GJB6* del(GJB6-D13S1830) deletion. Future studies should employ whole genome sequencing approaches and functional genomics studies to identify the other genes involved in most families, and in isolated cases of HI in Ghana.

## Data Availability

All datasets supporting the conclusions of this study are included in the manuscript and the **Supplementary Files**.

## Ethics Statement

The study was performed in accordance with the Declaration of Helsinki. Ethical approval was obtained from the Noguchi Memorial Institute for Medical Research Institutional Review Board, the University of Ghana, Accra (NMIMR-IRB-CPN 006/16-17 revd. 2018), and the University of Cape Town’s Human Research Ethics Committee, reference 104/2018. Written informed consent was obtained from all participants if they were 18 years or older, or from the parents/guardians with verbal assent from children, including permission to publish photographs.

## Author Contributions

Conceived and designed the experiments: GAA, GKA, AW. Performed the experiments: SA, OQ, NM, KM. Patients’ recruitment, samples, and clinical data collection and processing: SA, GKA, Analyzed the data: SA, AW; Contributed reagents/materials/analysis tools: GAA, VN, CK, AW. Wrote the paper: SA, GAA, VN, CK, AW. Revised and approved the manuscript: SA, OQ, GAA, GKA, KM, VN, CK, NM, AW.

## Funding

The study was funded by the Wellcome Trust, grant number 107755Z/15/Z to GAA and AW (co-applicant); NIH, USA, grant number U01-HG-009716 to AW, and the African Academy of Science/Wellcome Trust, grant, number H3A/18/001 to AW. The funders had no role in study design, data collection and analysis, decision to publish, or preparation of the manuscript.

## Conflict of Interest Statement

The authors declare that the research was conducted in the absence of any commercial or financial relationships that could be construed as a potential conflict of interest.

## Supplementary Material

Figure S1 Onset and time of HI test. **(A)** Age of deaf students at the first medical HI test. **(B)** Onset of HI. Paired T-test was used to compare the mean number of students with pre-lingual (n = 754) and post-lingual (n = 336) HI from 11 schools for the deaf. There was a significant difference between mean number of people with pre- and post-lingual HI with P value of 0.0001 (t = 7.68, df = 10).

Figure S2 Major causes of childhood HI in Ghana. **(A)** Major causes of post-lingual HI in Ghana. **(B)** Major causes of Pre-lingual HI in Ghana. Cerebrospinal meningitis was represented as C.S.M. The cause of HI labelled accident comprises of motor accidents and medical accidents such as wrong medication, child birth, and surgery. Diseases such as boil, anemia, Gilbertese, Jaundice, measles, mumps, Otitis media, and rubella were captured as other diseases while undefined sickness consist of individuals who developed the condition due to sickness, but the cause of the sickness was not determined.
