# Erratum: *GJB2* and *GJB6* Mutations in Non-Syndromic Childhood Hearing Impairment in Ghana

**DOI:** 10.3389/fgene.2019.01151

**Published:** 2019-11-26

**Authors:** Frontiers Production Office

**Affiliations:** Frontiers Media SA, Lausanne, Switzerland

**Keywords:** hearing impairment, genetics, *GJB2*, *GJB6*, Ghana, Africa

Due to a production error, the phrase “GJB2 (connexin 30)” should be “GJB6 (connexin 30).” Furthermore, the phrase “GJB2-D3S1830” should be “GJB6-D3S1830.”

A correction has been made to the **Abstract**:

“Our study aimed to investigate GJB2 (connexin 26) and GJB6 (connexin 30) mutations associated with non-syndromic childhood hearing impairment (HI) as well as the environmental causes of HI in Ghana. Medical reports of 1,104 students attending schools for the deaf were analyzed. Families segregating HI, as well as isolated cases of HI of putative genetic origin were recruited. DNA was extracted from peripheral blood followed by Sanger sequencing of the entire coding region of *GJB2*. Multiplex PCR and Sanger sequencing were used to analyze the prevalence of GJB6-D3S1830 deletion. Ninety-seven families segregating HI were identified, with 235 affected individuals; and a total of 166 isolated cases of putative genetic causes, were sampled from 11 schools for the deaf in Ghana. The environmental factors, particularly meningitis, remain a major cause of HI impairment in Ghana. The male/female ratio was 1.49. Only 59.6% of the patients had their first comprehensive HI test between 6 to 11 years of age. Nearly all the participants had sensorineural HI (99.5%; *n* = 639). The majority had pre-lingual HI (68.3%, *n* = 754), of which 92.8% were congenital. Pedigree analysis suggested autosomal recessive inheritance in 96.9% of the familial cases. *GJB2*-R143W mutation, previously reported as founder a mutation in Ghana accounted for 25.9% (21/81) in the homozygous state in familial cases, and in 7.9% (11/140) of non-familial non-syndromic congenital HI cases, of putative genetic origin. In a control population without HI, we found a prevalent of *GJB2*-R143W carriers of 1.4% (2/145), in the heterozygous state. No GJB6-D3S1830 deletion was identified in any of the HI patients. *GJB2*-R143W mutation accounted for over a quarter of familial non-syndromic HI in Ghana and should be investigated in clinical practice. The large connexin 30 gene deletion (GJB6-D3S1830 deletion) does not account for of congenital non-syndromic HI in Ghana. There is a need to employ next generation sequencing approaches and functional genomics studies to identify the other genes involved in most families and isolated cases of HI in Ghana.”

Additionally, in the **Results** section, the word “GJB2” should be italicized.

A correction has been made to the **Results**, subsection **Molecular Analysis Result of** ***GJB2*** **and** ***GJB6:***

“A total of 81 families segregating non-syndromic hearing loss were molecularly investigated. Although samples were not collected from Adamarobe, the ‘Deaf village,’ 27 out of the 81 HI families screened for *GJB2* and *GJB6* were from the Eastern Region of Ghana (Table S2) where the ‘Deaf Village’ is located (Kusters, 2012).One individual from each family was sequenced for *GJB2* mutation and we found a pathogenic mutation in 27.2% (22/81) with *GJB2*- R143W in the majority (21/22) in the homozygous state (Table 3); *GJB2* p.W44* mutation in one case, in the homozygous state.”

The original version of this article has been updated.

