# Supplementary figures and images for: GJB2 and GJB6 Mutations in Non-Syndromic Childhood Hearing Impairment in Ghana
Source: Front Genet. 2019 Sep 18;10:841. doi: 10.3389/fgene.2019.00841 (PMC6759689; doi:10.3389/fgene.2019.00841)

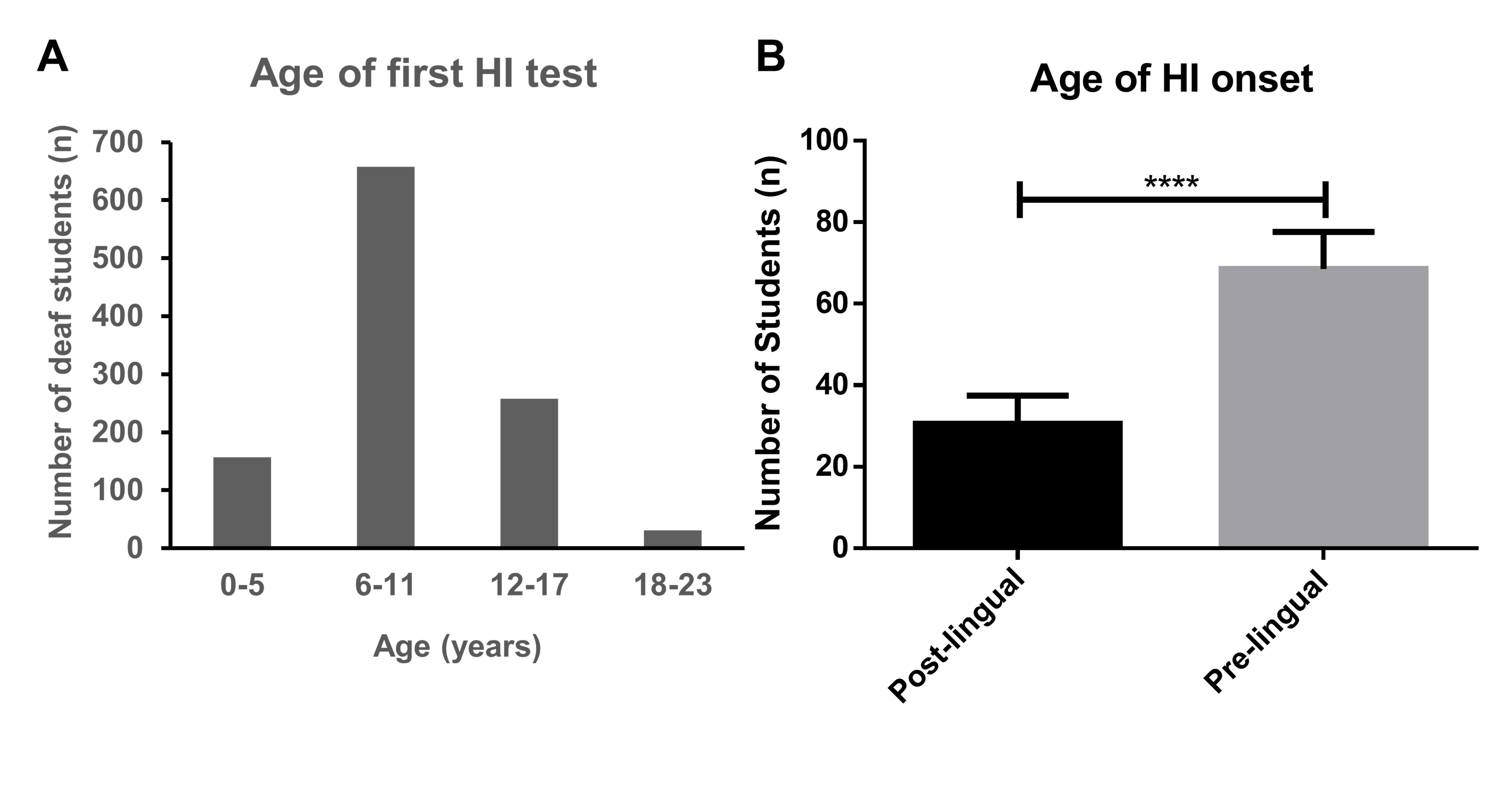

Supplement: Figure S1 — Onset and time of HI test. (A) Age of deaf students at the first medical HI test. (B) Onset of HI. Paired T-test was used to compare the mean number of students with pre-lingual (n = 754) and post-lingual (n = 336) HI from 11 schools for the deaf. There was a significant difference between mean number of people with pre- and post-lingual HI with P value of 0.0001 (t = 7.68, df = 10). [file Image_1.tif]

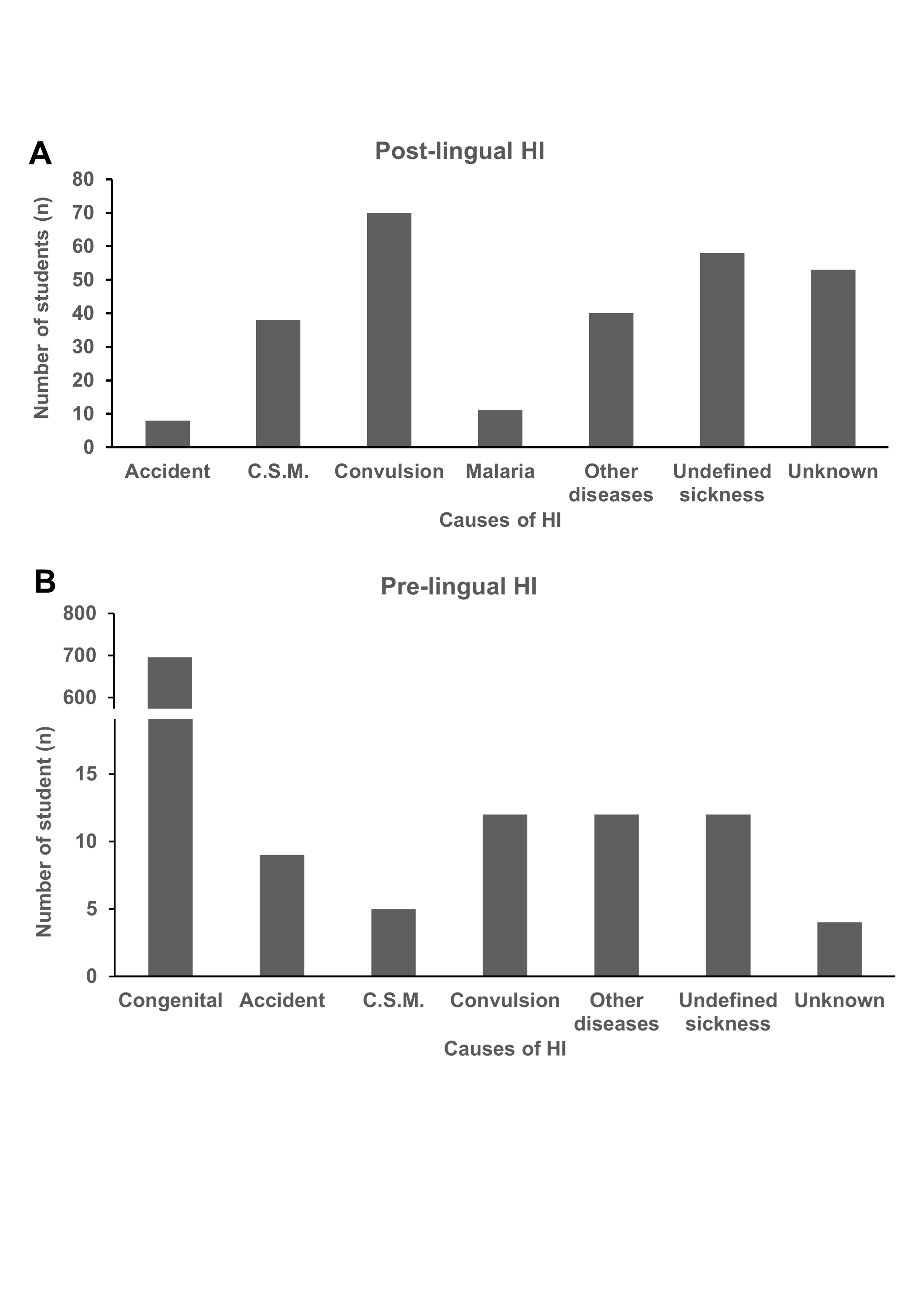

Supplement: Figure S2 — Major causes of childhood HI in Ghana. (A) Major causes of post-lingual HI in Ghana. (B) Major causes of Pre-lingual HI in Ghana. Cerebrospinal meningitis was represented as C.S.M. The cause of HI labelled accident comprises of motor accidents and medical accidents such as wrong medication, child birth, and surgery. Diseases such as boil, anemia, Gilbertese, Jaundice, measles, mumps, Otitis media, and rubella were captured as other diseases while undefined sickness consist of individuals who developed the condition due to sickness, but the cause of the sickness was not determined. [file Image_2.tif]
