# Supplementary material for: GJB2 and GJB6 Mutations in Non-Syndromic Childhood Hearing Impairment in Ghana
Source: Front Genet. 2019 Sep 18;10:841. doi: 10.3389/fgene.2019.00841 (PMC6759689; doi:10.3389/fgene.2019.00841)
Supplement: Supplementary file 3 [file Table_1.docx]

**Table S1: Categorization of HI based on degree of HI**

|  |  | **Number of students (%)** | |
| --- | --- | --- | --- |
| **Degree of HI** | **Category** | **Left ear** | **Right ear** |
| Mild-Moderate | 30-70 dB | 2 (0.31%) | 2 (0.31%) |
| Moderately-Severe | 71-90 dB | 6 (0.93%) | 6 (0.93%) |
| Severe | 91-100 dB | 34 (5.30%) | 37 (5.76%) |
| Severe-Profound | 101-110 dB | 28 (4.36%) | 27 (4.21%) |
| Profound | >120 dB | 572 (89.10%) | 570 (88.79%) |
| **Total** |  | **642 (100%)** | **642 (100%)** |
