# Supplementary material for: GJB2 and GJB6 Mutations in Non-Syndromic Childhood Hearing Impairment in Ghana
Source: Front Genet. 2019 Sep 18;10:841. doi: 10.3389/fgene.2019.00841 (PMC6759689; doi:10.3389/fgene.2019.00841)
Supplement: Supplementary file 4 [file Table_2.docx]

**Table S2: Geographical distribution of *GJB2* positive families in Ghana**

|  | Number of families (n) | | |
| --- | --- | --- | --- |
| Region/location | *GJB2* positive | *GJB2* negative | Total |
| Greater Accra | 1 | 5 | 6 |
| Ashanti | 5 | 10 | 15 |
| Central | 3 | 1 | 4 |
| Eastern | 7 | 20 | 27 |
| Northern | 1 | 2 | 3 |
| Upper East | 2 | 7 | 9 |
| Volta | 3 | 8 | 11 |
| Upper West | 0 | 6 | 6 |
| Total | 22 | 59 | 81 |
